# The relationships between health-related behaviours in the Canadian adult population

**DOI:** 10.1186/s12889-019-7674-4

**Published:** 2019-10-24

**Authors:** Adriana N. Mudryj, Natalie D. Riediger, Andrea E. Bombak

**Affiliations:** 1 Department of Food and Human Nutritional Sciences, Faculty of Food and Agricultural Sciences, University of Manitoba, 402 Human Ecology, Winnipeg, Canada; 2 Department of Community Health Sciences, Rady Faculty of Health Sciences, University of Manitoba, 750 Bannatyne Avenue, Winnipeg, Canada; 3 Department of Sociology, University of New Brunswick, Fredericton, Canada

**Keywords:** Health behaviour, Canadian health measures survey, Tobacco, Alcohol use, Fruit and vegetable, Physical activity, Sleeping habits, Diet

## Abstract

**Background:**

Health-related behaviours such as physical inactivity, low fruit and vegetable intake, smoking, alcohol use, and inadequate sleep are significant predictors of adverse health outcomes. Health promotion strategies often focus on one behavior, though research suggests health-related behaviours tend to co-occur. The purpose of this study is to describe the relationships between health-related behaviours in the Canadian adult population.

**Methods:**

Data from cycles 3 (2012–2013) and 4 (2014–2015) of the Canadian Health Measures Survey were pooled to describe health-related behaviours (current smoking status, high-risk alcohol use, fruit and vegetable intake, inadequate sleep, and physical activity) among adults according to sex, age group, household education, and income adequacy. Logistic regression was used to test for relationships between health-related behaviours.

**Results:**

Findings indicated that adverse health-related behaviours co-occur frequently, with approximately half of Canadians reporting two or more adverse health-related behaviours. Overall, Canadian men were more likely to report adverse health-related behaviours compared to women, with the exception of inadequate sleep. Smoking status, fruit and vegetable intake, sleep and physical activity exhibited an income and education gradient. Sex-based patterns in grouping of behaviours were present such that adverse health-related behaviours were associated with current smoking among men and with high-risk alcohol use among women.

**Conclusion:**

Our findings suggest that health-related behaviours should be considered in both *isolation* and *combination* when designing intervention strategies. Sex-specific patterns of how these behaviours co-occur must also be taken into account.

## Background

Health-related behaviours such as physical activity, fruit and vegetable intake, smoking, alcohol use, and sleeping habits are significant and, presumably, modifiable behavioural predictors of numerous health outcomes like type 2 diabetes, cardiovascular disease, and cancer [1]. Although health promotion strategies often focus on isolated health-related behaviors, many health-related behaviours typically do not occur as separate behaviours, but co-occur together [2]. Further, research in other countries suggests that the majority of individuals exhibit two or more adverse health-related behaviours [3], which aggregate more frequently in certain population subgroups [4].

Understanding the relationships among adverse health-related behaviours is important and may provide valuable information for designing appropriate intervention programs. For example, smoking has been shown to have the most bearing on other adverse health-related behaviours [5]. Smoking has been consistently associated with alcohol use at levels exceeding the national guidelines [6]; low intake of fruits and vegetables; significantly higher intakes of energy, fat, cholesterol, and alcohol; and lower intakes of fibre, polyunsaturated fats, and antioxidant vitamins [7]. Similarly, low physical activity has been associated with not eating fruit on the previous day as well as increased alcohol use among U.S. adolescents [8]. Research also suggests that men and individuals with low socioeconomic status are more likely to report co-occurring adverse health-related behaviours [4, 9].

The co-occurrence of health-related behaviours has exhibited geographic patterns according to neighbourhood socioeconomic status [10]. Social and economic inequities have a strong impact on health and well-being. The social determinants of health, including income, support networks, education, and colonialism influence health-related behaviours in a multitude of ways, affecting both mental and physical health [11]. Despite a growing body of literature examining the co-occurrence of health-related behaviours, there is little consensus about which behaviours occur together in certain sub-groups, for example, sex. Moreover, the co-occurrence of health-related behaviours may also differ across time and contexts. As such, a current examination in the Canadian context is needed. This evidence could inform current intervention and implementation strategies to address risk of non-communicable disease by identifying populations who report multiple adverse health-related behaviours, and examining the role of social determinants of health in influencing singular and combined health-related behaviours. Therefore, the purpose of this study is to describe health-related behaviours in the Canadian adult population according to age, sex, income adequacy, and household education, as well as describe the associations between health-related behaviours.

## Methods

### Study design and sample

This study used data from the Canadian Health Measures Survey (CHMS), cycles 3 (2012–2013) and 4 (2014–2015). The CHMS is a comprehensive, self-reported direct health measures survey that included blood, urine, and anthropometric measures, as well as a food frequency questionnaire (FFQ) [12]. Cycles 3 and 4 surveyed 5785 and 5794 respondents, with response rates of 51.7 and 53.7%, respectively. A full description of the household and individual response rates for each survey are fully described elsewhere [13, 14]. Participants < 18 years old and pregnant women were not included in our sample, excluding 2396 in cycle 3 and 2394 in cycle 4 (total sample excluded, *n* = 4790). A total of 6789 respondents met our inclusion criteria.

### Measures

Five self-reported health-related behaviours were included in the present study: current smoking, alcohol use, fruit and vegetable intake, inadequate sleep, and physical activity. *Smoking* was dichotomized as current, including occasional or daily, versus non –smoker (which included former smokers). *Alcohol use* was dichotomized into low and high-risk use, with high-risk defined as ≥5 drinks for men or ≥ 4 drinks for women on one occasion ≥2 times per month over the past year OR consuming alcohol every day in the past year [15]. *Fruit and vegetable intake*, obtained from the Food Frequency Questionnaire, was deemed adequate at ≥4 servings per day (excluding potatoes and juice) and inadequate when < 4 servings per day were consumed [16]. *Inadequate sleep* was dichotomized as ≤6 h/day (‘short duration’) or ≥ 10 h/day (‘long duration’) OR two or more of the following: having trouble going to sleep or staying asleep most or all of the time; never or rarely feeling refreshed by sleep; or finding it difficult to stay awake during normal waking hours when you want most or all of the time [17]. *Physical activity* was dichotomized as adequate and inadequate levels, based on an average of ≥30 min of low, moderate or vigorous physical activity on at least 5 days per week using results from an activity-monitoring device. Device data were blind to respondents while they wore it. Additionally, a “valid” day was defined as > 10 h of monitor wear time. Respondents with at least 4 valid days were retained for analyses in the CHMS [6]. The number of adverse health-related behaviours was summed for each respondent, providing a value between 0 and 5, and dichotomized as ≥3 adverse health-related behaviours and < 3 behaviours.

The following socioeconomic variables were included: sex, age group, highest level of education (household), and income adequacy. *Sex* was dichotomized as man and women; notably the CCHS did not have any other options. Given this omission, and the survey’s explicit use of the term sex, we consider this a measure of a respondent’s sex (i.e. their physiological and physical characteristics based on chromosomal complement) and not their gender (socially constructed roles and identities) [18]. *Age* was grouped as 18–29, 30–39, 40–49, 50–59 and ≥ 60 years. *Highest level of household education* was grouped as: less than secondary school graduation, secondary school graduation, and post-secondary graduation. *Income adequacy* was categorized into 4 groups as defined by Statistics Canada [19] based on total household income and number of individuals in the household, and was grouped as: lowest income group, lower middle income group, upper middle income group, and highest income group. Notably, Statistics Canada provides imputed values for household income due to the high percentage of missing values, as previously described [12].

### Analysis

All data analyses were performed using SPSS Statistics Software (IBM International) and STATA (StataCorp) and conducted in the secure location of the Manitoba Research Data Centre. Significance was set at *p* < 0.05. We conducted a sex and gender-based analysis (SGBA) where sample sizes allowed. Different disciplines have grappled with how to define, describe, and operationalize the complexity of sex and gender [20–22]. Our usage of sex and gender is in line with those proposed by the Canadian Institutes of Health Research [18] and the Institute of Medicine [23]. Sex differences refer to the physiological and biological differences between males and females at the cellular and organ levels, while a combination of social, identity-related, environmental and cultural influences contribute to gender differences. Briefly, a SGBA means considering both sex and gender in the analysis and interpretation of the results, including stratifying results by sex and considering both sex and gender in the interpretation of the findings [24]. While the CCHS data only includes a sex variable (not gender), many of the findings are interpreted primarily via socially-based gender roles.

Participant survey weights and the bootstrapping method were used in all the data analyses for this study. This approximation technique is recommended by Statistics Canada for use with the CHMS to estimate standard errors, coefficients of variation and confidence intervals, which apply to population-level estimates. The bootstrapping method was used to estimate the distributions from a sample’s statistics and involves the selection of random samples known as replicates, and the calculation of the variation in the estimates from replicate to replicate [25]. This technique also mitigates the effect of non-response. There were minimal missing data; as such, no additional techniques were used to account for missing data. Response rates for the health-related behaviours are as follows: current smoking (99%), high-risk alcohol use (83%), inadequate sleep (99%), fruit and vegetable intake (100%), and inadequate physical activity (90%).

First, health-related behaviours were described as proportions with standard error (SE), according to previously mentioned demographic factors. Chi-square tests were used to test for differences in each health-related behaviour according to sex, age group, education, and income adequacy. Second, we report number of adverse health-related behaviours. Third, we used logistic regression to test for predictors (age, sex, education, and income) of reporting ≥3 adverse health-related behaviours. Fourth, cross-tabulations were conducted for each combination of health-related behaviours. For example, we described the proportion of people who currently smoke who also report high-risk alcohol use, inadequate fruit and vegetable intake, low physical activity, or inadequate sleep. Finally, logistic regression was used to test for relationships of all health-related behaviours with each other. For example, all health-related behaviours with the exception of physical activity were examined as predictors of inadequate physical activity. Separate models for each sex resulted in ten models, or five health-related behaviours as outcomes (i.e. models) per sex.

Project approval was granted by Statistics Canada, which allowed project members to access the data. Research for this study was conducted at the Manitoba Research Data Centre and was consistent with Research Ethics Board Requirements. Data were analyzed in a secure environment and all output was vetted to prevent release of any identifying information.

## Results

### Health-related behaviours by demographic factors

Prevalence of reported health-related behaviours are summarized in Table 1. The most common health-related behaviour was low fruit and vegetable consumption (69.1%). Inadequate sleep was the second most common adverse health-related behaviour reported (36.0%), followed by high-risk alcohol use (26.6%), current smoking (22.1%), and low physical activity (17.9%). Overall, men were more likely to report adverse health-risk behaviours than women, with the exception of inadequate sleep. Household education and income adequacy were significantly associated with all health-risk behaviours, with the exception of high-risk alcohol use.

**Table 1:** Health-related behaviours according to demographic and socioeconomic variables

Demographic (n)	Current smoker	High-risk alcohol use^a^	Inadequate sleep	Inadequate physical activity	< 4 servings of fruit & vegetables per day	≥3 adverse behaviours
Sex						
Male (3360)	26.1 (1.2)	32.7 (1.8)	35.5 (1.5)	20.1 (1.4)	75.9 (1.4)	25.5 (1.3)
Female (3429)	18.2 (1.3)	20.3 (1.7)	36.5 (1.5)	15.8 (0.9)	62.3 (1.7)	16.8 (1.0)
*p*-value	< 0.001	< 0.001	0.619	0.006	< 0.001	< 0.001
Age (y)						
18–29 (1139)	26.3 (2.3)	31.8 (3.3)	34.4 (2.5)	26.8 (2.1)	73.4 (2.0)	26.3 (2.3)
30–39 (1443)	25.7 (3.6)	26.9 (3.3)	33.1 (2.5)	20.6 (2.5)	66.9 (2.6)	22.7 (2.1)
40–49 (1342)	24.4 (2.2)	24.6 (2.6)	35.2 (2.9)	16.9 (2.3)	67.4 (2.2)	22.9 (2.6)
50–59 (803)	22.3 (1.6)	24.8 (2.4)	43.6 (3.1)	14.6 (2.3)	69.9 (2.8)	20.7 (1.9)
60 + (2062)	13.2 (0.9)	24.6 (1.6)	34.1 (1.8)	11.3 (0.7)	67.3 (1.9)	13.0 (0.8)
p-value	< 0.001	0.178	0.057	< 0.001	0.165	< 0.05
Household Education						
< Secondary School (476)	40.0 (4.1)	33.0 (5.3)	47.9 (4.4)	29.5 (4.9)	80.3 (3.9)	38.0 (3.2)
Secondary School Graduation (951)	34.5 (3.5)	29.7 (3.2)	44.4 (3.4)	21.1 (2.3)	73.2 (2.3)	31.7 (3.3)
Post-Secondary School Graduation^b^ (5124)	18.1 (1.0)	25.2 (1.8)	33.1 (1.4)	16.0 (0.9)	67.4 (1.4)	17.0 (1.2)
p-value	< 0.001	0.168	< 0.01	< 0.01	< 0.01	< 0.001
Income Adequacy^c^						
Lowest (464)	42.6 (4.9)	32.2 (7.3)	39.6 (5.0)	27.9 (4.6)	77.0 (3.6)	32.9 (2.1)
Lower Middle (1051)	32.7 (3.2)	34.7 (3.4)	43.1 (2.9)	23.9 (2.6)	69.1 (3.4)	29.8 (1.9)
Upper Middle (1941)	23.0 (2.0)	27.0 (2.3)	37.9 (1.7)	16.9 (1.7)	72.3 (2,.0)	21.9 (1.9)
Highest (3333)	16.2 (1.2)	26.3 (1.8)	32.6 (1.7)	17.9 (1.5)	66.4 (1.3)	16.9 (1.8)
p-value	< 0.001	0.687	< 0.05	< 0.05	< 0.05	< 0.001

Note: SE = standard error

^a^High risk use was defined as ≥5 drinks (males) and ≥ 4 drinks (females) on one occasion ≥2 times per month over the past year OR consuming alcohol every day in the past year;

^b^Includes trade certificate or diploma, college, CEGEP or other non-university certificate or diploma, university certificate or diploma below the bachelor’s level, bachelor’s degree, university certificate/diploma/degree above the bachelor’s level

^c^Classifies total household income into 4 categories based on total household income and the number of people living in the household

### Number of co-occurring health-related behaviours

**Fig. 1:**
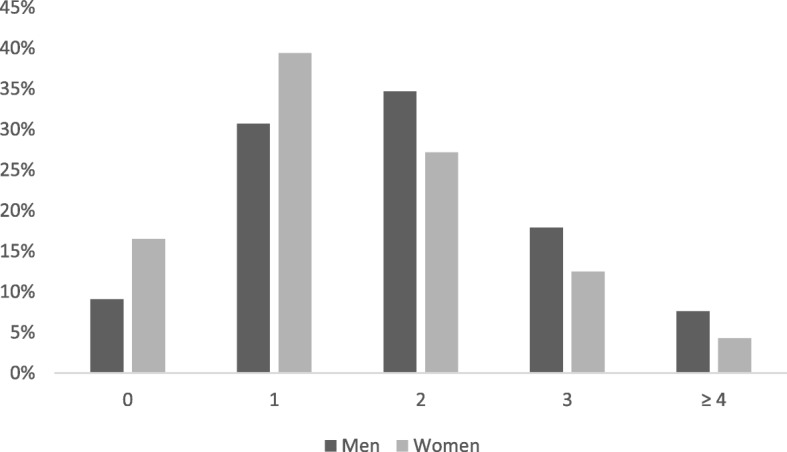
Co-occurring health-related behaviours (%)

Overall, 30.9% of Canadians reported two adverse health-related behaviours, and 21.2% report three or more adverse behaviours. One-quarter of men reported three or more adverse health-related behaviours compared to 16.8% of women (Fig. 1). Specifically, women were 40% less likely to report ≥3 adverse health-related behaviours compared to men (*p* < 0.01), independent of age group, education, and income (Table 2**)**. Younger age, lower level of education, and lower income adequacy were also significantly associated with increased odds of reporting ≥3 health-related behaviours.

**Table 2:** Odds ratio (95% CI) of ≥3 adverse health-related behaviours

	Men and women	Men	Women
Sex			
Men	Reference		
Women	0.61 (0.48–0.76)**		
Age (y)			
18–29	Reference	Reference	Reference
30–39	0.84 (0.58–1.22)	1.05 (0.60–1.85)	0.64 (0.33–1.27)
40–49	0.96 (0.63–1.46)	1.26 (0.66–2.39)	0.67 (0.40–1.13)
50–59	0.61 (0.42–0.89)*	0.62 (0.36–1.07)	0.63 (0.36–1.12)
≥ 60	0.36 (0.24–0.54)**	0.49* (0.26–0.93)	0.22 (0.14–0.35)***
Household Education			
< Secondary School	Reference	Reference	Reference
Secondary School Graduation	0.60 (0.36–1.01)	0.76 (0.41–1.39)	0.44 (0.16–1.25)
Post-Secondary School Graduation	0.30 (0.20–0.46)*	0.43 (0.24–0.75)**	0.19 (0.07–0.47)**
Income Adequacy			
Lowest	Reference	Reference	Reference
Lower Middle	0.83 (0.46–1.48)	0.77 (0.33–1.73)	0.90 (0.32–2.50)
Upper Middle	0.67 (0.38–1.20)	0.69 (0.28–1.68)	0.64 (0.22–1.84)
Highest	0.52 (0.31–0.86)*	0.52 (0.27–0.99)*	0.49 (0.18–1.38)

*p < 0.05; **p < 0.01; ***p < 0.001

### Association of health-related behaviours

Respondents who smoked demonstrated the highest proportions of inadequate sleep, low physical activity, high-risk alcohol use, and low fruit and vegetable consumption as compared to respondents who reported any other adverse health-related behaviour (Table 3). For example, while the overall prevalence of high-risk alcohol use was 26.0%, among current smokers, 38.2% reported high-risk alcohol use.

**Table 3:** Co-occurring health-related behaviours (% (SE))

Health-related Behaviour	Smoking (22.1 (1.0))	High-risk alcohol use (22.0 (1.4))	Inadequate sleep (36.0 (1.1))	Inadequate physical activity (17.9 (0.9))	Low fruit and vegetable intake (69.1 (1.3))
Smoking (1309)	–	38.2 (3.2)	46.7 (2.0)	26.6 (2.7)	77.4 (2.0)
High-risk alcohol use (1408)	33.2 (2.0)	–	40.0 (2.6)	19.3 (1.8)	73.4 (1.8)
Inadequate sleep (2363)	28.6 (1.9)	30.3 (2.5)	–	20.2 (1.5)	68.8 (2.1)
Inadequate physical activity (1194)	32.8 (3.7)	28.6 (3.2)	40.5 (2.6)	–	74.4 (2.7)
Low fruit and vegetable intake (4655)	24.7 (1.4)	28.1 (1.7)	35.9 (1.5)	19.3 (1.0)	–

Note: SE, Standard Error

For men, smoking was a significant predictor of all other health-risk behaviours. Other than smoking, no other health-related behaviours were significantly associated with each other, independent of other health-related behaviours among men. Among Canadian women, high-risk alcohol use was associated with increased odds of smoking, inadequate sleep, low physical activity and low fruit and vegetable consumption (Table 4). Among women, smoking was only significantly associated with high-risk alcohol use.

**Table 4:** Odds of health-related behaviours according to other health-related behaviours (OR (95% CI))

Health-related behaviour (predictor)	Outcome				
	Smoking	High-risk alcohol consumption	Inadequate sleep	Inadequate physical activity	Low fruit and vegetable intake
Men					
Smoking	–	1.89 (1.27–2.81)**	1.77 (1.23–2.56)**	1.53 (1.07–2.21)**	1.56 (1.02–2.39)*
High-risk alcohol consumption	1.89 (1.27–2.81)**	–	1.22 (0.87–1.71)	1.22 (0.87–1.71)	0.98 (0.75–1.28)
Inadequate sleep	1.77 (1.23–2.56)**	1.22 (0.87–1.71)	–	0.94 (0.71–1.23)	1.09 (0.77–1.54)
Inadequate physical activity	1.53 (1.07–2.21)**	0.98 (0.75–1.28)	0.94 (0.71–1.23)	–	1.11 (0.73–1.67)
Low fruit and vegetable intake	1.56 (1.02–2.39)*	0.97 (0.70–1.46)	1.09 (0.77–1.54)	1.11 (0.73–1.67)	–
Women					
Smoking	–	2.06 (1.19–3.56)*	1.12 (0.70–1.78)	0.95 (0.56–1.61)	1.31 (0.90–1.89)
High-risk alcohol consumption	2.06 (1.19–3.56)*	–	1.68 (1.12–2.53)*	2.44 (1.45–4.09)**	1.54 (1.01–2.34)*
Inadequate sleep	1.12 (0.70–1.78)	1.68 (1.12–2.53)*	–	1.57 (1.07–2.31)*	0.83 (0.63–1.09)
Inadequate physical activity	0.95 (0.56–1.61)	2.44 (1.45–4.09)**	1.57 (1.07–2.31)*	–	1.44 (1.04–2.00)*
Low fruit and vegetable intake	1.31 (0.90–1.89)	1.54 (1.01–2.34)*	0.83 (0.63–1.09)	1.44 (1.04–2.00)*	–

*p < 0.05; ** < 0.01

## Discussion

The current study identified discernible patterns of health-related behaviours in the Canadian adult population. Findings indicated that about 21% of Canadian adults report at least three adverse health-behaviours, which is consistent with other research in the general Canadian adult population [3]. Overall, a higher proportion of Canadian men reported all adverse health-related behaviours compared to women, with the exception of inadequate sleep. This is in contrast to previously published work, which suggests that women are at an increased risk of sleep disorders, including insomnia and lower quality sleep, and that sleep dysregulation may have more severe health consequences for women [26]. Sex-based patterns in groupings of health-related behaviours were present such that adverse behaviours co-occurred more strongly with current smoking among men and with high-risk alcohol use among women.

Fruit and vegetable intake, inadequate sleep, smoking, and inadequate physical activity all demonstrated an income and education gradient, consistent with other Canadian studies [5, 27]. The pathways between socioeconomic status and the various health-related behaviours are multiple and complex [28]. Low-income neighbourhoods may be less likely to have facilities or locations such as parks, gyms or community centres that facilitate physical activity. Families may also shift towards cheaper, more energy-dense foods when incomes drop, often-forgoing high quality proteins, fruits, and vegetables [29]. Although it is not clear whether lower income leads to shorter, lower quality sleep or vice versa, research suggests the correlation between poverty and sleep does exist [30, 31].

The consistent and significant association between socioeconomic status and several health-related behaviours, and *how they occur together*, underscores the necessity of addressing social determinants of health. Consideration of these associations could potentially maximize effective, targeted interventions in low socioeconomic groups, subsequently reducing the adverse health effects of these behaviours. The need to address social determinants is further supported by growing evidence of the limited tractability of many health-related behaviours [32–34].

Alcohol use has shown a two-way relationship with socioeconomic status; risky or heavy alcohol use has been shown to predict unemployment, and unemployment increases the odds of problem alcohol use among young men in the UK [35]. Furthermore, lower lifetime income trajectories were associated with higher odds of both adult alcohol abstinence and heavy drinking in US adults [36]. The lack of socioeconomic gradient for high-risk alcohol use in the present study may be specific to the Canadian context and time period, or it may reflect the criteria used to dichotomize high- and low-risk alcohol use. Notably, high-risk alcohol use in Canada has increased over time [37].

Results from our study also reveal sex differences in the co-occurrence of health-related behaviours. High-risk alcohol use in women demonstrated increased odds of all other adverse health-related behaviours examined in this study. This pattern did not hold true for men. To interpret these differences and conduct a SGBA [18] it is important to discuss sex and gender, and their respective influences on health-related behaviours. The sex differences observed may be related to the rise in binge drinking among young Canadian women [37], which may be driven by a number of social factors. For example, relatively recently women have experienced increased participation in the workforce and subsequently increased income [38], and cultural norms related to alcohol use for women have changed such that it is more acceptable for women to binge-drink [39]. Importantly, women are more likely to consume alcohol in response to negative emotions and stress as compared to men [40], but women also view alcohol as an important and pleasurable aspect of their social life [41].

The increasing rates of alcohol-related hospitalization of Canadian women [42] and recently published recommendations that deem no amount of alcohol is safe [43], suggest that further research examining drinking among Canadian women is needed. We have previously reported that Canadian women who report high-risk alcohol use also report better self-rated health [44], suggesting future avenues of research should focus on the sociological aspects to explain why women drink alcohol. A particular focus on Canadian women in the workforce may be warranted given the lack of socioeconomic gradient in high-risk alcohol use and the potential role of stress in driving these relationships between health-related behaviours.

For men, smoking was a significant predictor of all other health-related behaviours, which was not the case for women. Similar to the relationships observed with high-risk alcohol use among women, it is important to consider both sex and gender [45] in the interpretation of relationships of health-related behaviours and smoking among men. The Surgeon General’s Report concluded that women who smoke are more susceptible to depression and anxiety disorders than non-smokers, and that women trying to quit smoking relapse for different reasons than men [45]. Women are more likely to use smoking as a coping mechanism for stress, weight control and negative emotions, while men who smoke do so more for stimulation and in pleasurable settings [46]. Smoking is strongly associated with alcohol use among men [47], which is, at least partially, due to increased pleasure from smoking cigarettes when consuming alcohol [48]. Neuroimaging studies also suggest that smoking activates men’s reward pathways more than women’s, consistent with the idea that men smoke for the reinforcing effects of nicotine [47]. Taken together, these results suggest both sex and gender differences, i.e. social differences, may be affecting different relationships between smoking and other health-related behaviours among men and women.

The different patterns of relationships between health-related behaviours, particularly concerning smoking or high-risk alcohol use, suggest pleasure and behaviors associated with sociability remain strong influencers of behavior, regardless of public health recommendations. Public health, when urging behavior modification or abstinence, must grapple with the legitimate value of pleasure in individuals’ lives, particularly when considering issues of health equities [49, 50].

### Limitations

The study is subject to limitations. The pooling of samples from two time periods has limitations, namely if a large change has occurred in the sample populations, for example age structure. Given the close time period of data collection between the two surveys though, any change is likely to be fairly minimal. Importantly, the survey questions that were used in the present study did not change between the two surveys. Research that is dependent upon voluntary subject participation is particularly vulnerable to sampling bias. Notably, the CHMS has a low response rate, which may have impacted the analysis reported here, specifically in terms of underestimating adverse health-related behaviours. Response bias of self-assessed behaviour has been observed in the literature, potentially resulting in underestimation in prevalence of health-related behaviours [52]. Unfortunately, we are unable to test for differences among respondents and those who did not participate, which limits our ability to speculate as to how non-response may have influenced relationships between health-related behaviours. The lower response rate for the questions related to alcohol use is also a limitation and may have influenced the results. Finally, all health-related behaviours have been dichotomized. Each health-related behaviour has demonstrated a dose response relationship or J-shaped relationship with a variety of health outcomes. Regardless of threshold effects, some information may have been lost through dichotomizing, such as grouping former smokers with never smokers in the non-smoker group.

## Conclusions

Health-related behaviours are important because they are linked cumulatively to morbidity and mortality. Our results suggest that adverse health-related behaviours should be considered in both isolation and combination when designing health promotion policies, and further reiterates the importance of addressing social determinants of health. The gender-specific co-occurrence of health-related behaviours (i.e. drinking in women; smoking in men) both suggest that social interactions or pleasure may be fueled by behaviours that have a detrimental effect on the health of Canadians. A greater emphasis must be placed on the social function, interactions, and meanings of tobacco and alcohol. Affirmative, salutogenic approaches to health and structural, gender-specific interventions that facilitate positive mental and social health and alternative sources of pleasure may be necessary. Results of this study provide useful data to inform the creation and evaluation of health promotion strategies in order to achieve maximum positive health impact by individual, group and population-level approaches.

## Data Availability

The datasets used and analyzed during the current study are available by permission from Statistics Canada and the Canadian Research Data Centre Network.

## Abbreviations

CHMS: Canadian Health Measures Survey
SGBA: Sex and gender based analysis
